# Circular RNA hsa_circ_0000523 regulates the proliferation and apoptosis of colorectal cancer cells as miRNA sponge

**DOI:** 10.1590/1414-431X20187811

**Published:** 2018-11-01

**Authors:** Y. Jin, L.L. Yu, B. Zhang, C.F. Liu, Y. Chen

**Affiliations:** 1 Department of General Surgery, Tongde Hospital of Zhejiang Province, Zhejiang, China; 2 Department of Anesthesiology, the Children's Hospital, Zhejiang University School of Medicine, Zhejiang, China

**Keywords:** Circular RNA hsa_circ_0000523, Colorectal cancer, Proliferation, Sponge, Interference

## Abstract

Among the novel class of endogenous long non-coding RNAs, circular RNA (circRNA) is known as a key regulator in the development and progression of different cancers. Its function and mechanism in the tumorigenesis of colorectal cancer, however, has not been well studied. This study thus aimed to investigate potential regulation of colorectal cancer by circRNAs and the corresponding regulatory mechanism. We demonstrated that the expression of circRNA hsa_circ_0000523 (also known as circ_006229) was down-regulated in different colorectal cancer cell lines. It was also found that interference of hsa_circ_0000523 induced proliferation and suppressed apoptosis of colorectal cancer cells, the proliferation rate of which was reduced by the overexpression of hsa_circ_0000523. In addition, we found that miR-31 could recognize hsa_circ_0000523 sequence and that it acted as a “sponge” of miR-31, indirectly regulating Wnt/β-catenin signaling pathway, which was involved in the progression of colorectal cancer. The results suggested that the expression of hsa_circ_0000523 correlated to the tumorigenesis of colorectal cancer cells. In addition, as a sponge of miR-31, the low level of hsa_circ_0000523 led to activation of Wnt/β-catenin signaling pathway, inducing the subsequent progress of colorectal cancer.

## Introduction

Colorectal cancer has the third highest incidence rate and fourth highest mortality rate among all cancer cases (1). Although common treatments of colorectal cancer such as surgical resection, radiotherapy, chemotherapy or their combination extend life expectancy for up to 5 years, mortality and recurrence rates are high. It is, therefore, important to develop new molecular biomarkers and targets for more effective treatments.

Circular RNAs (circRNAs) are a novel class of endogenous long non-coding RNAs with their 3′- and 5′-ends joined together to form a covalently closed loop (2 [Bibr B03] [Bibr B04]). Most eukaryotic circRNAs present in cytoplasm and are produced by trans-splicing exons of pre-mRNAs or non-coding transcripts of coding genes (2–6). CircRNA has recently attracted much research interest due to its wide expression found in many genes (7–9). It was found that circRNAs functioned as microRNA (miRNA) sponges (5,10–18) and were involved in the initiation and progression of different cancers (14 [Bibr B15] [Bibr B16] [Bibr B17],^19^–21). Li and co-workers reported that circRNA, hsa_circ_002059 was significantly down-regulated in gastric cancer tissues compared with the non-tumorous tissues of the same origin and that the down-regulation varied with distal metastasis, TNM stage, gender, and age, suggesting that circRNAs could be used as potential biomarkers for diagnosis of gastric carcinoma (19). The regulating mechanism of circRNA in cancer was investigated in several studies: in esophageal squamous cell carcinoma (ESCC), cir-ITCH acted as sponge of miRNAs to both increase the level of ITCH and inhibit the Wnt/β-catenin pathway, therefore inhibiting ESCC (14); in breast cancer cells (MDA-MB-231), circ-Foxo3 (hsa_circRNA_104170) bonded with 8 miRNAs (miR-22, miR-136, miR-138, miR-149, miR-433, miR-762, miR-3614-5, and miR-3622b-5p) to promote the translation of Foxo3 mRNA, thus inhibiting cell proliferation (22). Bachmayr-Heyda and colleagues observed a globally lower expression of circRNAs in colorectal cancer cells compared to their healthy counterparts; a negative correlation between the circRNA abundance and the proliferation of colorectal cancer cells was also determined by the team (23).

Based on Bachmayr-Heyda's study, we focused on hsa_circ_0000523 (also known as circ_006229), which was down-regulated in colorectal cancer tissues and cell lines, to further explore the function of circRNA in colorectal cancer. The expression of hsa_circ_0000523 was also negatively correlated with the proliferation rate among different colorectal cell lines (23).

## Material and Methods

The study was approved by Animal Ethics Committee of Tongde Hospital of Zhejiang Province, and the experiments with rats were in full compliance with the European Communities Council Directive of 24 November 1986 (86/609/EEC) or with the Guidelines laid down by the NIH (USA).

### Cell culture and transfection

Cells were seeded into 6-well plates at a density of 2×10^5^ cells/well for siRNA (Genepharma, China) or miRNA mimic (Ribobio, China) transfection. Transfection was performed using Lipofectamine 3000 (Invitrogen, USA), following the manufacturer's instructions. Final concentration of transfections was 100 nM. Sequences of siRNAs were (sense strands are reported as 5′- 3′): si-circ_0000523-1: GAGCAAGAAGAUCUACGGAdTdT; siControl-1: GAGCAAGAAGUAGAUGCCUdTdT; si-circ_0000523-2: GAAGAUCUACGGAAUCCAGAdTdT; siControl-2: CUUCAUCUACGGAAUCCAGAdTdT; si-circ_0000523-3: CAACAGAGCAAGAAGAUCUAdTdT; siControl-3: CAACAGAGCAAGAAGUAGAUdTdT (Table 1).

**Table 1:** Growth medium used for each cell line.

Cell line	Growth medium
COLO205, COLO320HSR, DLD-1, HCT-15, HCT-8	RPMI-1640
SW480, SW620, SW1116	Leibovitz's L-15
HT-29	McCoy's 5A (Modified)
LoVo	F-12K
NCM460	Dulbecco's Modified Eagle's Medium (DMEM)
FHC	DMEM/F12^a^
LS 174T	Eagle's Minimum Essential Medium (EMEM)^b^
Caco-2	EMEM^c^

^a^Supplemented with HEPES (10 mM), cholera toxin (10 ng/mL), insulin (0.005 mg/mL), transferrin (0.005 mg/mL), and hydrocortisone (100 ng/mL); ^b^supplemented with 10% fetal bovine serum, penicillin (100 U/mL), and streptomycin (100 μg/mL); ^c^supplemented with 20% fetal bovine serum, penicillin (100 U/mL), and streptomycin (100 μg/mL).

### RNA isolation and qPCR assay

Total RNA was isolated using TRIzol reagent (Invitrogen). Complementary DNA (cDNA) was reversely transcribed using TransScript reverse transcriptase (Transgene) with random primers. Primer pairs for qPCR were designed using Primer-BLAST (https://www.ncbi.nlm.nih.gov/tools/primer-blast). Primers were checked for specificity by gel, melting curve analysis, and sequencing. miRNA primers were ordered from Ribobio (China). qPCR cycling was initially performed at 95°C for 2 min, then 40 cycles at the conditions of 95°C/15 s, 60°C/15 s, and 68°C/20 s. The relative expression of each target gene was determined using the formula 2^−△△Ct^. GAPDH was used as the internal control while parallel negative control experiments were performed in the absence of cDNA. Sequences of primers were: circ-F: CAGCATCGGAACCAGCAAAG; circ-R: CTGGGCTGTCACTACGGAAG; GAPDH-F: GCTCTCTGCTCCTCCTGTTC; GAPDH-R: ACGACCAAATCCGTTGACTC.

### Dual luciferase assay

A literature vector (24) was employed as a reporter of miRNA by inserting the potential target sequence in frame to form fused reporter gene with the target site located in the coding region. The inhibitory effect of miRNA on the target sequence represented the effectiveness of miRNA recognizing the target. The DNA oligos of miRNA targets were synthesized and cloned into the vector. The reporter vector (100 ng/well) with the target site of the tested miRNA, the pRL-TK control vector (50 ng/well), and miRNA or negative control (NC) mimics (20 nM in each case) were transfected into the HEK293A cells. The luciferase activities were determined with a Dual-Luciferase Reporter Assay System (Promega, USA) following the manufacturer's protocol.

### Western blot

Cells were harvested, washed with phosphate buffered saline (PBS), and lysed with lysis buffer (Sigma, USA). The bicinchoninic acid (BCA) protein assay (Invitrogen) was used to determine the protein concentration of cell lysate. The nucleoprotein was extracted using Nuclear and Cytoplasmic Protein Extraction Kit (Beyotime Biotechnology, China) following the product specification. The resulting proteins (50 mg) were loaded for electrophoresis on 10% Tris-glycine polyacrylamide gels and transferred onto polyvinylidene fluoride membranes. The membranes were blocked in 2.5% BSA for 1 h and incubated with the primary antibodies (β-catenin (Abcam, 1:1000 dilution), Dkk1 (Abcam, 1:1000 dilution), and c-Myc (Abcam, 1:1000 dilution) or β-actin (Bioworld, 1:4000 dilution)) at 4°C overnight, and then with the second antibody (ZSGB-Bio, 1:4000 dilution) at room temperature for 2 h after rinsing. Western blot was detected using a chemiluminescence detection system (CWBIO).

### Cell proliferation assay

Cells were seeded into 96-well plates at a density of 3×10^3^ cells/well and incubated for 24 h, after which the medium was removed, and the cells were washed with PBS. Pre-diluted CCK-8 (1:10, 100 μL/well) was added to the cells, which were incubated at 37°C for 2 h. Intensity of absorbance was determined using a Multimode Reader (BioTek, USA).

### Cell apoptosis assay

Apoptosis was assessed using an ApoDETECT annexin V-FITC apoptosis detection kit (Sigma-Aldrich) by flow cytometry following the manual. Cells were digested, washed twice with cold PBS, and re-suspended in binding buffer. FITC-labeled annexin V (5 μL) was added to the cell suspension (190μL) and mixed, followed by the addition of propidium iodide (PI) solution (5 μL). Cells were incubated in the dark at room temperature for 10 min and were then analyzed on a flow cytometer (BD Biosciences, USA).

### Statistical analysis

Data are reported as means±SE. Single-factor differences between two sets of data were analyzed for statistical significance by Student's *t*-tests. Multiple comparisons were analyzed with ANOVA followed by the Bonferroni *post hoc* test using GraphPad Prism software (USA).

## Results

### CircRNA hsa_circ_0000523 was down-regulated in colorectal cancer cell lines

**Figure 1:**
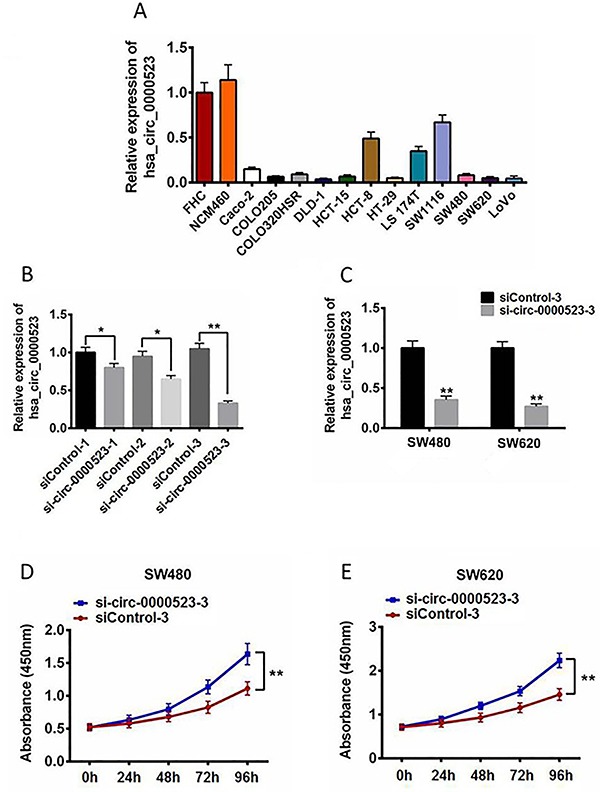
*A*, Expression profile of hsa_circ_0000523 in 12 different human colorectal cancer cell lines and 2 human normal intestinal cell lines (FHC and NCM460). *B*, hsa_circ_0000523 expression levels in FHC cells after transfection with 3 siRNAs targeting hsa_circ_0000523 and corresponding control siRNAs. CircRNA expression levels were assessed by qPCR and normalized to GAPDH. *C*, hsa_circ_0000523 expression levels in SW480 and SW620 cells transfected with si-circ_0000523-3 and siControl-3. Cells were harvested after 24 h. CircRNA expression levels were assessed by qPCR and normalized to GAPDH. Cell viability of SW480 (*D*) and SW620 (*E*) were assessed using the CCK8 assay after siRNA transfection (100 μM). The experiments were performed in triplicate independently. Data are reported as means±SE. *P<0.05; **P<0.01 (ANOVA or *t*-test).

It was previously found that RNA-seq showed a global reduction of circRNA abundance in both colorectal cancer cell lines and tissues (23). In order to investigate the function of circRNA during the development of colorectal cancer, circRNA hsa_circ_0000523 was selected as a potential regulator in colorectal cancer. To understand its expression profile in colorectal cancer cells, expression levels of hsa_circ_0000523 in 12 different human colorectal cancer cell lines and 2 human normal intestinal cell lines were assessed using qRT-PCR. Lower expression of hsa_circ_0000523 was observed in all tested cancer cell lines compared with the normal ones (Figure 1A): expression level of hsa_circ_0000523 in most cancer cell lines (Caco-2, COLO205, COLO320HSR, DLD-1, HCT-15, HT-29, SW480, SW620, LoVo) was only 15% or less relative to normal intestinal cell lines (FHC, NCM460), while that in HCT-8, LS 174T, and SW1116 cells was approximately half compared to their healthy counterparts. The results demonstrated a reduced expression of hsa_circ_0000523 in 12 different human colorectal cancer cell lines, suggesting that there might be a correlation between the down-regulation of hsa_circ_0000523 and the development of colorectal cancer.

### Hsa_circ_0000523 regulated the proliferation of colorectal cancer cells

To study if hsa_circ_0000523 was involved in the development of colorectal cancer, RNAi assay was performed to inhibit the expression of hsa_circ_0000523. Three siRNAs targeting hsa_circ_0000523 were designed and their silence efficiencies were tested on FHC cells (Figure 1B). Results showed that all three siRNAs down-regulated hsa_circ_0000523 significantly in FHC cells with si-circ_0000523-3 the most efficient. Si-circ_0000523-3 was thus transfected into SW480 and SW620 cells, followed by the assessment of both the expression level of hsa_circ_0000523 and the proliferation of SW480 and SW620 cells. It was found that the expression level significantly decreased by approximately 70% in SW480 and SW620 cells (Figure 1C) and that the proliferative ability of SW480 and SW620 cells significantly improved posterior to the si-circ_0000523-3 transfection (Figure 1D and E). The results showed that inhibition of hsa_circ_0000523 expression facilitated the growth of colorectal cancer cells, suggesting that expression of hsa_circ_0000523 might regulate colorectal cancer development.

**Figure 2:**
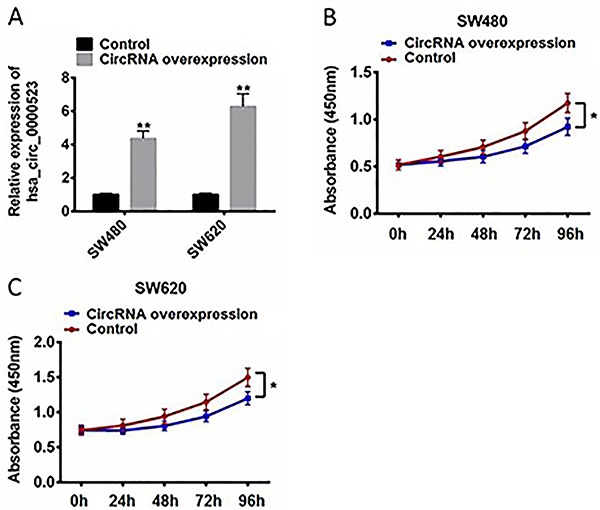
*A*, hsa_circ_0000523 expression levels in SW480 and SW620 cells after hsa_circ_0000523 overexpression using lentiviral expression systems. CircRNA expression levels were assessed by qPCR and normalized to GAPDH. Cell viability of SW480 (*B*) and SW620 (*C*) were assessed using CCK8 assay after hsa_circ_0000523 overexpression. The assays were performed in triplicate independently. Data are reported as means±SE. *P<0.05. **P<0.01 (ANOVA or *t*-test).

To further explore the function of hsa_circ_0000523, overexpression assay was also performed in colorectal cancer cells. Overexpression plasmids were constructed by inserting hsa_circ_0000523 cDNA into pLVX-IRESneo. hsa_circ_0000523 was up-regulated approximately 4-fold in SW480 and 6-fold in SW620 (Figure 2A). In addition, the overexpression of hsa_circ_0000523 inhibited the proliferation of both SW480 and SW620 cells (Figure 2B and C). The results showed that hsa_circ_0000523 had an inhibitory effect on the proliferation of colorectal cancer cells, consistent with the results from the hsa_circ_0000523 inhibition experiment. A conclusion that could be made from the gain-of-function and loss-of-function assays was that the expression of hsa_circ_0000523 regulated the proliferation of both SW480 and SW620 cells.

### Hsa_circ_0000523 induced cell apoptosis in colorectal cancer cells

**Figure 3:**
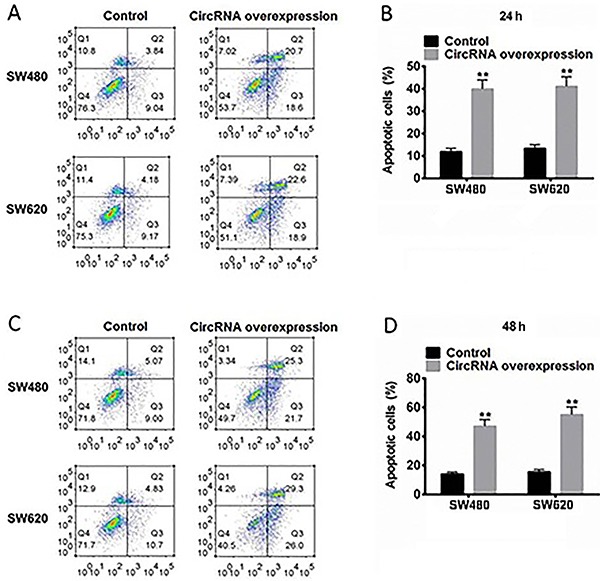
Hsa_circ_0000523 induced apoptosis in human colorectal cancer cells. *A* and *C*, Representative images of flow cytometry analysis in SW480 and SW620 cells. The early and late apoptosis was quantified and indicated in Q3 and Q2 gates, respectively. Percentage of apoptotic cells after 24 h (*B*) and 48 h (*D*). Data are reported as means±SE from three independent experiments. **P<0.01 (*t*-test).

To explore the possible mechanism for hsa_circ_0000523 regulating proliferation of colorectal cancer cells, the effect of hsa_circ_0000523 on apoptosis of colorectal cancer cells was hence measured by flow cytometry. The rate of apoptosis was significantly increased in hsa_circ_0000523-overexpressed SW480 and SW620 cells after 24 h (Figure 3A and B) and both early and late apoptosis rates (Figure 3A and C) and the total apoptosis rate (Figure 3B and D) increased slightly after 48 h compared with that after 24 h. The results demonstrated that hsa_circ_0000523 inhibited colorectal cancer cell proliferation, by potentially inducing cell apoptosis.

### Hsa_circ_0000523 regulated proliferation of colorectal cancer cells via miR-31

**Figure 4:**
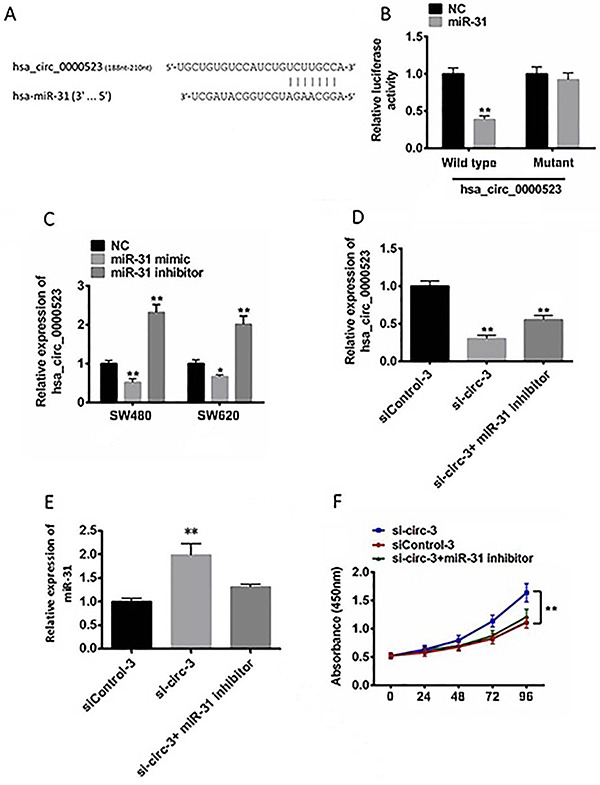
*A*, Schematic representation of miR-31 and predicted target site in hsa_circ_0000523. *B*, HEK293A cells were co-transfected with reporter carrying the predicted target of miR-31 in hsa_circ_0000523 and the corresponding small RNA, assessed using a dual-luciferase reporter assay system and compared to normal control (NC) transfection. *C*, hsa_circ_0000523 expression levels in SW480 and SW620 cells after transfection with miR-31 or miR-31 inhibitor. *D*, hsa_circ_0000523 expression levels in SW480 cells after transfection with si-circ_0000523-3 or co-transfection of si-circ_0000523-3, and miR-31 inhibitor. *E*, miR-31 expression levels in SW480 cells after transfection with si-circ_0000523-3 or co-transfection of si-circ_0000523-3, and miR-31 inhibitor. CircRNA and miRNA expression levels were assessed by qPCR and normalized to GAPDH or U6. *F*, Cell viability of SW480 assessed using CCK8 assay after transfection with si-circ_0000523-3 or co-transfection of si-circ_0000523-3 and miR-31 inhibitor. The assays were performed in triplicate independently. Data are reported as means±SE. *P<0.05; **P <0.01 (ANOVA).

A major function of circRNAs is sponging miRNAs. It was hence predicted there might be miRNAs that could recognize sequences in hsa_circ_0000523 and interact with it. Based on the results of TargetScan, we found that several miRNAs could potentially recognize targets in hsa_circ_0000523, such as miR-31, miR-558, and miR-1270. After preliminary screening by miRNA mimics transfection, miR-31 was chosen as a candidate for further studies, for the inhibition effect of miR-31 mimics on hsa_circ_0000523 (pre-experiment data not shown). The predicted target sequence of miR-31 in hsa_circ_0000523 is shown in Figure 4A: the 2-8 nt of miR-31, the “seed-region”, perfectly matched the predicted target in the circRNA. Such a match was known to be decisive to miRNA target recognition (25,26), therefore miR-31 was considered able to recognize and bind to hsa_circ_0000523 specifically.

To study the interaction between hsa_circ_0000523 and miR-31, target recognition efficiency of miR-31 was assessed using dual-luciferase system. Both wild type and mutated target sequences in hsa_circ_0000523 were inserted into the coding region of luciferase gene separately, and then co-transfected modified reporter plasmids and miR-31 mimics into HEK293A cells. It was shown that miR-31 significantly down-regulated the expression of luciferase with wild type target, but not with mutated target (Figure 4B). The results confirmed that miR-31 recognized the predicted target in hsa_circ_0000523.

In addition, the effect of miR-31 on endogenous hsa_circ_0000523 was assessed using miR-31 mimic and miR-31 inhibitor. Transfection with miR-31 mimic moderately down-regulated hsa_circ_0000523 in both SW480 and SW620 cells, while transfection with miR-31 inhibitor significantly up-regulated hsa_circ_0000523 for 2.0–2.3-fold (Figure 4C). The results were consistent with the finding described above.

We further investigated the expression level of miR-31 in SW-480 cells after transfection of si-circ_0000523-3. The expression level of miR-31 doubled that of hsa_circ_0000523 and reduced by 70% by siRNA (Figure 4D and E), compared with the control group. This suggested that hsa_circ_0000523 negatively regulated miR-31 in SW-480. The proliferation of SW480 was found to be promoted by si-circ_0000523-3 transfection (Figure 4F). Upon the co-transfection of si-circ_0000523-3 and miR-31 inhibitor, the level of hsa_circ_0000523 was down-regulated compared with the control (Figure 4D) and the si-circ_0000523-3 transfection-induced proliferation was rescued by miR-31 inhibitor (Figure 4F). The results showed that there was interaction between hsa_circ_0000523 and miR-31 and that they could interdependently regulate each other to reach a kinetic balance in colorectal cancer cells. The inhibitor of miR-31 rescued the inductive effect of si-circ_0000523-3 on colorectal cancer cell proliferation, indicating that hsa_circ_0000523 regulated the growth of colorectal cancer cells via the interaction with miR-31.

### Hsa_circ_0000523 regulated Wnt/**β**-catenin signaling pathway via miR-31

**Figure 5:**
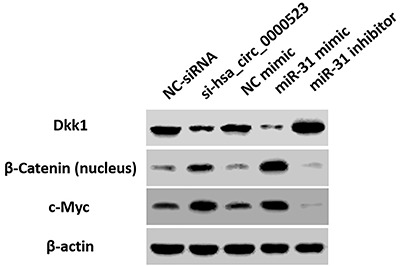
Hsa_circ_0000523 and miR-31 regulated the Wnt/β-catenin pathway. Western blot analysis of Dkk1, β-Catenin in nucleus, and c-Myc expression in SW480 cells transfected with si-circ_0000523, miR-31 mimic, miR-31 inhibitor individually. β-actin was used as internal control.

It was reported that miR-31 promote breast tumorigenesis by suppressing Wnt signaling antagonists, Dkk1 (27). Therefore, we investigated if the expression level of hsa_circ_0000523 and miR-31 related to Wnt/β-catenin signaling pathway. Compared with the NC siRNA transfection, si-circ_0000523-3 transfection decreased the protein level of Dkk1 but increased those of β-Catenin in nucleus and c-Myc (Figure 5). Considering that down-regulation of hsa_circ_0000523 would induce up-regulation of miR-31, miR-31 mimic was transfected in SW480. miR-31 mimic also down-regulated DDK1 level and activated the Wnt/β-catenin signaling pathway in SW480 cells (Figure 5). miR-31 inhibitor suppressed Wnt/β-catenin signaling pathway. The findings indicated that hsa_circ_0000523 suppressed the Wnt/β-catenin signaling pathway via sponging miR-31, which acted as an activator of this pathway, which is involved in the progression of colorectal cancer.

## Discussion

The function of circRNA remains unclear to this date (28,29), however previous studies showed that circRNA acted as regulators and valuable biomarkers of diseases, including cardiovascular diseases and multiple tumors (30). In this study, hsa_circ_0000523 was found to be down-regulated in colorectal cancer cell lines. Further analyses were conducted to determine the biological function and molecular mechanism of hsa_circ_0000523 in the development of colorectal cancer.

By performing loss-of-function and gain-of-function assays, we demonstrated that hsa_circ_0000523 not only inhibited the proliferation of SW480 and SW620 cells but also induced apoptosis of colorectal cancer cells. This finding suggested that the down-regulation of hsa_circ_0000523 contributed to the development of colorectal cancer, both by inducing abnormal proliferation and by suppressing cell apoptosis.

It was previously reported (31–33) that circRNA could act as a miRNA “sponge” to absorb functional miRNAs, decreasing the abundance of miRNA in the cytoplasm. Thus, we considered that hsa_circ_0000523 might absorb miRNAs as well and miR-31 was chosen for the research. miR-31 recognized and bound hsa_circ_0000523 and the expression level of hsa_circ_0000523 negatively regulated that of miR-31 level. Transfection of miR-31 mimic down-regulated hsa_circ_0000523 but transfection of miR-31 inhibitor up-regulated it. Co-transfection of si-circ_0000523-3 and miR-31 inhibitor induced limited the rescuing effect caused by siRNA, indicating that miR-31 and hsa_circ_0000523 interdependently regulated each other in colorectal cancer cells. In terms of cell proliferation, miR-31 inhibitor was found to reverse the increase in the proliferation rate caused by low expression of hsa_circ_0000523, suggesting that hsa_circ_0000523 regulated proliferation of colorectal cancer cells via miR-31. We also observed that the co-transfection led to a mild increase of miR-31 at the same time. miRNA inhibitor blocked the function of miRNA by binding with homologous miRNA but did not induce the degradation of miRNA. These blocked miRNAs could still be detected by qRT-PCR. In addition, the reduction in expression of hsa_circ_0000523 resulted in more released miR-31. As a result, co-transfection of si-circ_0000523-3 and miR-31 inhibitor slightly up-regulated miR-31.

It was previously reported that hsa_circ_0000523 promotes breast tumorigenesis by suppressing DDK1, an antagonist of Wnt signaling pathway (27). Thus, we assessed the expression level of DDK1 and factors related to Wnt/β-catenin signaling pathway and found that Wnt/β-catenin pathway was activated by transfecting miR-31 mimic and suppressed by miR-31 inhibitor. This finding showed that both down-regulation of hsa_circ_0000523 and up-regulation of miR-31 could activate Wnt/β-catenin signaling pathway in colorectal cancer cells. Considering the interdependent regulation between hsa_circ_0000523 and miR-31, such an activation/suppression relationship suggested that hsa_circ_0000523 regulated Wnt/β-catenin signaling pathway via absorbing miR-31, influencing the proliferation and apoptosis process of colorectal cancer cells. Down-regulation of hsa_circ_0000523 in colorectal cancers disrupted the dynamic balance between it and miR-31, thus leading to a consequent activation of Wnt/β-catenin signaling pathway, which then promoted the tumorigenesis of colorectal cancer cells.

This study demonstrated that circRNA hsa_circ_0000523 was down-regulated in 12 colorectal cancer cell lines and that it negatively regulated proliferation of colorectal cancer cells via sponging miR-31. In addition, the decrease in the expression of hsa_circ_0000523 was involved in the tumorigenesis of colorectal cancer through releasing miR-31, followed by the subsequent activation of Wnt/β-catenin signaling pathway.
